# Brexpiprazole mitigates hyperprolactinaemia induced by paliperidone in a patient with a history of skin reaction to aripiprazole: case report introducing a new therapeutic approach

**DOI:** 10.1192/bjo.2026.11052

**Published:** 2026-05-18

**Authors:** Marie Humbert-Claude, Tiphaine Barbé, Maria-Cristina Ionescu, Mehrnoush Khasian, Omar Timothy Khachouf, Anne-Laure Blanc, Delphine Lemoullec-Tourtier, Nicolas Widmer

**Affiliations:** Pharmacy of the Eastern Vaud Hospitals, Rennaz, Switzerland; Psychiatric Hospital, Fondation de Nant, Corsier-sur-Vevey, Switzerland; Institute of Pharmaceutical Sciences of Western Switzerland, University of Geneva, University of Lausanne, Geneva, Switzerland; Service of Clinical Pharmacology, Lausanne University Hospital, Lausanne, Switzerland

**Keywords:** Brexpiprazole, hyperprolactinaemia, antipsychotic-induced adverse effects, dopamine partial agonist, cross-reactivity

## Abstract

Hyperprolactinaemia is a common adverse effect of antipsychotic medication, primarily resulting from dopamine D2 receptor blockade. It is characterised by menstrual irregularities, gynaecomastia, galactorrhoea and, with prolonged exposure, an increased risk of osteoporosis and breast cancer. When switching towards a prolactin-sparing antipsychotic is not feasible, adjunctive use of aripiprazole – a dopamine D2 partial agonist – has emerged as a validated strategy to mitigate hyperprolactinaemia without compromising antipsychotic efficacy. However, alternative options are needed for patients who cannot tolerate aripiprazole. Here we report the use of brexpiprazole, a dopamine D2 partial agonist structurally related to aripiprazole, to counteract paliperidone-induced hyperprolactinaemia effectively in a patient who developed a cutaneous reaction to aripiprazole. During the follow-up period with adjunctive brexpiprazole in combination with paliperidone, neither a recurrence of psychotic symptoms nor a reappearance of the skin reaction previously experienced with aripiprazole was observed.

Hyperprolactinaemia is a common adverse effect of antipsychotic medication, with reported prevalence reaching up to 47% in women and around 18% in men treated with antipsychotics.^1^ The agents most commonly implicated are those with high dopamine D2 potency molecules,^2^ such as the first-generation antipsychotics along with amisulpride, risperidone and paliperidone; the last of these has the higher risk ratio for prolactin elevation according to a recent meta-analysis.^3^ The pathophysiological mechanism involves the blockade of dopamine D2 receptors located on the membrane of lactotroph cells in the anterior pituitary gland, which are the primary targets of the tubero-infundibular pathway, resulting in the loss of dopaminergic inhibition of prolactin secretion.^4^

Hyperprolactinaemia can lead to a spectrum of clinical symptoms, including headache, sexual dysfunction, menstrual disturbances, gynaecomastia and galactorrhoea. Of particular concern, long-term exposure (>5 years) to prolactin-raising antipsychotics may result in an increased risk of osteoporosis and breast cancer.^5^

## Adjunctive aripiprazole for managing antipsychotic-induced hyperprolactinaemia

When switching towards a prolactin-sparing antipsychotic is not feasible (such as in cases of intolerance, history of poor response or poor adherence), the adjunctive use of aripiprazole has emerged as a validated strategy to mitigate hyperprolactinaemia without compromising antipsychotic efficacy.^6^ Following a first report,^7^ several studies have confirmed that the addition of aripiprazole can reduce hyperprolactinaemia when induced by risperidone^8^ (including the long-acting injection form),^9^ paliperidone^10^ and haloperidol.^11^ However, its efficiency is reduced when it is used with amisulpride.^12,13^ Aripiprazole is a potent dopamine D2 partial agonist that stimulates D2 receptors to approximately 60% of the maximal effect of dopamine (i.e. 60% of intrinsic activity). This high level of intrinsic activity results in a decreased prolactin level in monotherapy,^14–16^ and appears sufficient to restore the inhibitory dopaminergic tone on prolactin secretion suppressed by another antipsychotic agents.

## Comparative prolactin-sparing effects between brexpiprazole and aripiprazole

Brexpiprazole, a novel atypical antipsychotic structurally related to aripiprazole (see Table 1), also exhibits partial agonist activity at D2 receptors, but with lower intrinsic activity compared with aripiprazole (approximately 43%). Consequently, brexpiprazole has less impact on serum prolactin levels than aripiprazole.^3,15,17^ In a retrospective study of youths with psychiatric disorders, aripiprazole was associated with hypoprolactinaemia whereas brexpiprazole significantly increased prolactin levels.^16^ Nevertheless, this increase remained minimal, and brexpiprazole is considered a prolactin-sparing antipsychotic similar to quetiapine, clozapine, cariprazine or aripiprazole. Accordingly, in a study evaluating the effect of brexpiprazole on prolactin in patients with schizophrenia, prolactin-related adverse effects occurred in 1.8% for brexpiprazole-treated patients versus 0.6% on placebo, leading the authors to propose that brexpiprazole could have potential as a prolactin-stabilising agent in schizophrenia, but that more research was needed before clinical implementation.^18^ Indeed, lower intrinsic activity of brexpiprazole at D2 receptors – compared with aripiprazole – introduces a reasonable pharmacological uncertainty regarding its capacity to reverse established hyperprolactinaemia.

**Table 1:** Comparative activity on the human dopamine D2 receptor

Pharmacological properties	Aripiprazole	Brexpiprazole
Chemical structure	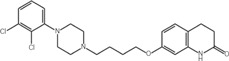	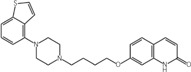
*K*i^25^	0.34 nM	0.30 nM
Activity on dopamine receptor D2	Partial agonist	Partial agonist
Intrinsic activity on hD2L^a^ ^25^	61%^26^	43%^27^

hD2L, human dopamine D2 receptor long isoform; *K*_i_, equilibrium constant, i.e. nanomolar amount of the antipsychotic needed to occupy 50% of the human dopamine D2 receptor in vitro.

a. The precise value may be debated; intrinsic activity can vary from 20 to 90% depending on in vitro assay.^26,27^

Despite the favourable prolactin profile of brexpiprazole when used as monotherapy, its potential to reverse hyperprolactinaemia induced by other antipsychotic agents – particularly paliperidone, the most prolactin-elevating agent^3,15^ – remains to be addressed.

Here we present a case illustrating the off-label use of brexpiprazole in reducing paliperidone-induced hyperprolactinaemia in a patient who experienced a skin reaction to aripiprazole.

## Case report

Ms M is a young adult female, aged 26 years and suffering from paranoid schizophrenia and cannabis addiction, who was admitted to hospital for a psychotic episode and remained hospitalised for 1 month. In the weeks before admission she presented with insomnia and persecutory and grandiose delusions, accompanied by auditory hallucinations. Episodes of agitation and conflict occurred with her family, linked to cannabis use. On the day of admission her speech was characterised by logorrhoea and thought processes were disorganised. Despite these clinical features, she did not exhibit significant agitation or hostility during her stay. She did not report any suicidal ideation.

Before admission she had undergone multiple antipsychotic trials. Given the patient’s history of poor adherence, the selection of a long-acting injectable antipsychotic with combined antipsychotic and mood-stabilising properties was required. Paliperidone treatment had been initiated at a low dose during out-patient follow-up, and gradually increased in hospital with the patient’s agreement, up to a dosage of 12 mg daily. The other medication prescribed was hydroxyzine 25 mg at bedtime and lorazepam 1 mg four times daily as required. The patient showed rapid clinical improvement, with a reduction in hallucinations and delusional thoughts along with improved therapeutic engagement and greater awareness of illness. Twenty-six days after treatment initiation a satisfactory plasma paliperidone level of 42 ng/mL was achieved (therapeutic range 20–60 ng/mL).^19^ However, Ms M developed galactorrhoea 4 weeks following paliperidone initiation. Two serial prolactin measurements, at weeks 4 and 5 after treatment onset, revealed persistent elevation of prolactin above the upper limit of normal, at 130 μg/L (physiological range 5–23 μg /L).

Because of the positive clinical response, the continuation of paliperidone was deemed appropriate by the medical team, a decision supported by the patient. Given the established efficacy of adjunctive aripiprazole for treating antipsychotic-induced hyperprolactinaemia, the addition of aripiprazole at low dose was considered. However, Ms M reported a prior skin reaction apparently due to aripiprazole, characterised by a cutaneous eruption. During hospitalisation a cautious challenge was attempted (2.5 mg/day, increased at 5 mg/day on day 3) but, again, 1 week later this resulted in a reproducible disabling skin eruption, puffy face and newly reported drowsiness and sedation, confirming intolerance and precluding the further use of aripiprazole. In light of these constraints, and given the similarities with aripiprazole, adjunctive low-dose brexpiprazole (1 mg) was introduced as an off-label treatment to counteract hyperprolactinaemia. Brexpiprazole has an elimination half-life of approximately 90 h, leading to steady-state concentration being reached at around 20 days.^20^

Three weeks following the introduction of brexpiprazole at 1 mg, a prolactin assay demonstrated a marked reduction in serum prolactin, from 130 to 37 μg/L (normal range 5–23 μg/L) and the galactorrhoea resolved. One month later the prolactin level remained at 39 μg/L. Notably, the patient’s periods subsequently returned to a regular pattern. The patient did not develop any skin reaction but complained of somnolence. Importantly, there was no recurrence or exacerbation of psychotic symptoms during this period.

## Discussion

This case report describes the successful introduction of brexpiprazole to counteract hyperprolactinaemia induced by paliperidone, without recrudescence of psychotic symptoms and without reappearance of the skin reaction previously experienced with aripiprazole.

### Absence of reaction with brexpiprazole following aripiprazole-induced skin rash

Aripiprazole-induced cutaneous reactions, such as rash or urticaria, are rare but have been reported in both the literature^21–24^ and product information. The aetiopathogenesis is largely unknown. Brexpiprazole is a derivative of aripiprazole with an added thiophene ring (see Table 1). To our knowledge there is no evidence of immunological cross-reactivity between aripiprazole and brexpiprazole, despite their structural similarities. In the present case, the patient experienced a reproducible cutaneous reaction to aripiprazole but tolerated brexpiprazole without any hypersensitivity reaction. This observation, if replicated by others, may indicate that the risk of cross-reactivity between these two agents may be low regarding skin reaction. This would be a potentially valuable consideration for clinical practice in cases where aripiprazole intolerance is a barrier to treatment.

### Intrinsic activity at dopamine D2 receptors and implications for hyperprolactinaemia

Brexpiprazole, like aripiprazole, is a potent dopamine D2 partial agonist but with lower intrinsic activity at this receptor (43%; see Table 1). The reduction of antipsychotic-induced hyperprolactinaemia relies on the restoration of dopaminergic tone at the D2 receptor, counteracting the prolactin-elevating effects of D2 antagonists. However, the minimal level of intrinsic D2 activity required to achieve a clinically meaningful reduction in prolactin remains to be determined. Moreover, some reports, albeit rare, have described hyperprolactinaemia induced by aripiprazole^28^ and brexpiprazole.^29^ Thus, it was uncertain whether brexpiprazole would effectively lower prolactin in this context. In the case of our patient, adjunctive brexpiprazole led to a significant decrease in prolactin levels without a resurgence of psychotic symptoms, indicating that its potency (equilibrium constant (*K*_i_) = 0.30 *v*. 1.6 nM for paliperidone),^30^ and its partial agonist activity, were sufficient to counteract the effect of paliperidone on D2 receptors localised on the lactotroph cells of the pituitary gland. We could also have tried cariprazine, the third available partial agonist, which has a high affinity for the D2 receptor (*K*_i_ = 0.49 nM)^25^ but displays even lower intrinsic activity than brexpiprazole (∼30%).^31^

### Normalisation of prolactin

Spontaneous normalisation of prolactin over time without the introduction of brexpiprazole cannot be entirely ruled out. Indeed, partial tolerance during chronic treatment may cause prolactin levels to trend towards normalisation. Nevertheless, elevated prolactin levels persist in most patients despite prolonged exposure to neuroleptic. Accordingly, prolonged exposure to prolactin-increasing antipsychotics has been associated with a decline in bone mineral density and the development of osteoporosis,^1^ and may elevate the risk of breast cancer.^5^ Furthermore, symptoms such as galactorrhea, gynaecomastia, menstrual irregularities, infertility and sexual dysfunction can be distressing for the individual. Consequently, treatment may be indicated with the aim of minimising both hyperprolactinaemia and the symptomatic burden.

To our knowledge, the off-label use of brexpiprazole to mitigate paliperidone-induced hyperprolactinaemia has not previously been described. If this finding is replicated by others, it may be clinically useful for managing antipsychotic-induced hyperprolactinaemia in patients who are intolerant to aripiprazole and for whom switching to a prolactin-sparring molecule is not possible.
